# Plant Microbe Interactions in Post Genomic Era: Perspectives and Applications

**DOI:** 10.3389/fmicb.2016.01488

**Published:** 2016-09-26

**Authors:** Jahangir Imam, Puneet K. Singh, Pratyoosh Shukla

**Affiliations:** Enzyme Technology and Protein Bioinformatics Laboratory, Department of Microbiology, Maharshi Dayanand UniversityRohtak, India

**Keywords:** plant–microbe interactions, plant immune response, beneficial interactions, genome-scale metabolic modeling, emerging pathogens, PHI-base

## Abstract

Deciphering plant–microbe interactions is a promising aspect to understand the benefits and the pathogenic effect of microbes and crop improvement. The advancement in sequencing technologies and various ‘omics’ tool has impressively accelerated the research in biological sciences in this area. The recent and ongoing developments provide a unique approach to describing these intricate interactions and test hypotheses. In the present review, we discuss the role of plant-pathogen interaction in crop improvement. The plant innate immunity has always been an important aspect of research and leads to some interesting information like the adaptation of unique immune mechanisms of plants against pathogens. The development of new techniques in the post - genomic era has greatly enhanced our understanding of the regulation of plant defense mechanisms against pathogens. The present review also provides an overview of beneficial plant–microbe interactions with special reference to *Agrobacterium tumefaciens*-plant interactions where plant derived signal molecules and plant immune responses are important in pathogenicity and transformation efficiency. The construction of various Genome-scale metabolic models of microorganisms and plants presented a better understanding of all metabolic interactions activated during the interactions. This review also lists the emerging repertoire of phytopathogens and its impact on plant disease resistance. Outline of different aspects of plant-pathogen interactions is presented in this review to bridge the gap between plant microbial ecology and their immune responses.

## Introduction

**FIGURE 1:**
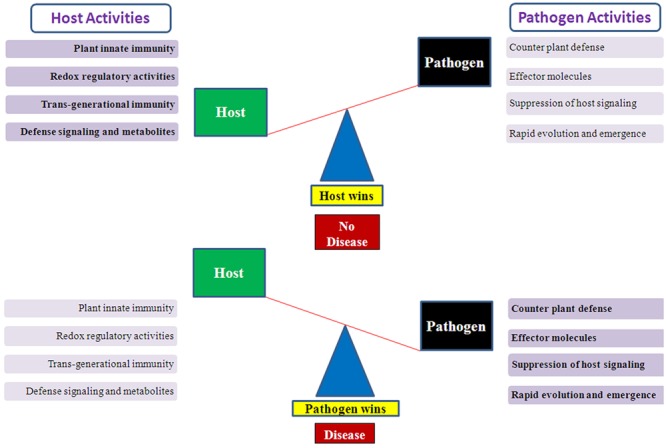
**Illustration showing host–pathogen activities which determine the host wins or pathogen wins situation.** A conceptual diagram showing the balance mechanism between host–pathogens activities. When host activities suppress the pathogen effect then host wins and plants survive the pathogen attack but when pathogen defeat the host defense system then pathogen wins and disease occurs.

The associations between plants and microbes/pathogens are highly diverse as the latter thrive and flourish below the ground, above the ground as well as within the plants (Vorholt, 2012; Bulgarelli et al., 2013). The microbes and their interactions can be both endophytic and epiphytic, and also with the nearby environment and soil in the vicinity of plant roots. The interaction between plant and microbes can be fruitful or beneficial, neutral, and unfavorable which directly influences the plant growth, its health, and development (Newton et al., 2010). A Single plant species acts as a host for only limited numbers of microbes/pathogens, and vice-versa. This degree of specialization and specificity leads to a high level of diversity among the microorganisms and their evolution over millions of years (Galagan et al., 2005). But many times the interaction of microbes with plants can be pathogenic and leads to infection in the plants (Strange and Scott, 2005). Earlier also, it was thought that pathogens and their degree of virulence can be both normal as well as an epidemic and causes severe yield losses in crop production and represent a major threat to global food security (**Figure 1**). There are many questions left to understand the plant-pathogen interactions better, which ultimately affects the health of the plants. Both commensal and pathogenic interaction require specific signaling pathways for mutual benefit interactions or disease responses like the development of root nodules or rice blast disease infection (Riely et al., 2004, Riely et al., 2006; Imam et al., 2014a, 2015a,b).

DNA and RNA-based studies, genomics data analyses, transcriptomics, metagenomics, metabolomics, next generation sequencing (NGS) technologies and proteomics approaches have proved to be valuable tools to study plant-pathogen interactions and their associations. Thus, plant-pathogen associations can now be studied at a speed and depth as never before (See Vocabulary). For the development of effective disease management strategies, the study of plant-pathogen interactions at much faster pace is the need of the hour (Madden and Wheelis, 2003). Global warming and climate change have also started showing adverse effects on defense systems in plants (Schenk et al., 2008). Since the interactions between different host and pathogens are generally similar over the wide range of species, the study and information on plant-pathogen interactions will enhance our ideas and perspective of wide-range of interactions (Sexton and Howlett, 2006; Torto-Alalibo et al., 2009). In this review, perspectives on the potential biological interactions to enlighten the processes between plants and pathogens that can be exploited for biotechnological applications are taken into consideration.

## Relevance of Plant-Microbe Interactions for Crop Improvement

Due to constant changing climatic conditions and global warming, crop yield and production has been severely affected and therefore the plant production system should be optimized for higher yield in the limited fertile land. To increase crop yield and production, the microbial/biological exploitation is the better solution and will play an important role in disease dissemination and control (Reid, 2011). Mainly the plant–microbe interaction research has been focused on three aspects, the oldest symbiosis between plants and mycorrhizae (Smith and Smith, 2011), nitrogen fixation in plants (Oldroyd et al., 2011) and pathogenesis (Dodds and Rathjen, 2010; Kachroo and Robin, 2013; Wirthmueller et al., 2013). These systems are now well characterized and provide insights into common and diverged signaling network mechanism in plant-pathogen interactions. Resistant crop breeding which uses the molecular breeding and genetic engineering approach to transfer the resistance genes or QTLs against pathogens is one of the better and most effective and environmentally friendly approach to counter microbial diseases as against the use of pesticides (Akhon and Machray, 2009; Gust et al., 2010). The exploitation of biotic and abiotic situation of plants is another environment friendly approach in developing sustainable disease management strategies (Haggag et al., 2015). However, the biotic approach requires a superior comprehension of how plants and microorganisms intimately interact with each other in a great degree of complex environment and how these interactions result in physiological changes in plants. Moreover, information is required on how plants organize their needs like utilizing energy and resources for their protection and resistance against the pathogens at the cost of their own growth and development (Haggag et al., 2015).

With the advent of NGS technologies which results in the completion of genome sequencing and re-sequencing of the over-whelming numbers of plant and their pathogens generating huge amount of data, we are witnessing an era of genomics and post-genomics with a challenge to translate these plethora of information for the crop improvements with broader disease resistance spectra (Knief, 2014). In the post-genomic era, the translational genomics presented a better solution in crop improvement against pathogenic bacteria, fungi, and viruses and prepare these crops in current thwarting climatic conditions (Knief, 2014). Now a days proteomics in combination with bioinformatics and computational biology are widely used methodology to decipher plant-pathogen interactions which are based on the idea of isolation, characterization and identification of whole set of proteins taking part in the process inside a cell under specific conditions at a particular time (Wilkins et al., 1995; Imam et al., 2014b). The proteome-level study provides insight into the real molecules which are involved in mediating specific cellular processes (Kav et al., 2007). Plant-pathogen proteomics is now in fact a challenge to scientists all over the world as the interactions involve proteins of both plant and microbes which is a difficult task to study and characterize (Mathesius, 2009). For the improvement of crop plants with the use of biotechnological approach, the involvement and information of key proteins are important which engage in proper growth and development of plants. The proteins involved are important for the maintenance of cellular functions in the plants by controlling physiological and biochemical pathways. Recent research in the post-genomic era showed that both genomics and proteomics are two important sides for the discovery of new genes which will be helpful in many crop improvement programs (Baggerman et al., 2005; Lambert et al., 2005; Scherp et al., 2011; Jayaraman et al., 2012).

## The Plant Immune System: An Insight Into Defense Mechanism

For many organisms like bacteria, fungi, protists, and insects, the richest source of nutrients is plants. Even though plants lack a proper immune system as present in animals but they have developed unique ways of defense mechanism at different levels like structural, chemical and protein-based to identify the pathogens and prevent further damage. Understanding how plants defend themselves from pathogens is essential in order to develop highly disease-resistant plant species. As plants are lacking in mobile immune cells and the cellular adaptive immune systems, they are mainly dependent on innate immunity and efficient signaling mechanisms (Dangl and Jones, 2001; Ausubel, 2005; Chisholm et al., 2006). The entry of pathogens into the plant cells is the first and most important step in disease response. Different pathogens have different mechanisms of penetration into the plant cells. Bacteria enter into the plant cells through trichomes, lenticels, stomata and other openings, fungi uses a specialized structure called hyphae and formation of penetration peg while viruses can only enter into the plant cells through physical injuries (Layne, 1967; Fox et al., 1971; Getz et al., 1983; Mendgen et al., 1996).

Once the pathogen breaks the primary defense barriers, mainly two branches of the plant immune response is elicited, namely, microbial (or pathogen) associated molecular patterns (MAMPs/PAMPs) triggered immunity (MTI/PTI) formerly called as basal or horizontal immunity and effector-triggered immunity (ETI) formerly called *R*-gene-based or vertical immunity. Plants have other modes of immune responses, like systemic acquired response (SAR) and gene silencing (Lu et al., 2003; Durrant and Dong, 2004; Padmanabhan et al., 2009; Sahu et al., 2012). The MTI/PTI immune response formerly known as basal or horizontal immunity is triggered when the pathogens release the elicitors called microbial-associated molecular patterns (MAMPs) which is recognized by the pattern recognition receptors (PRRs), a class of plasma-membrane bound extracellular receptors and gets activated which in turn results in active defence response and stop the further colonization and proliferation of infection (Hammond-Kosack and Jones, 1997; Hammond-Kosack and Parker, 2003; Dodds and Rathjen, 2010; Beck et al., 2012). Pathogen-associated molecular patterns (PAMPs) which activate innate immune responses in animals also mediate the activation of plant defense. Furthermore, structurally similar recognition complexes as same as to animal PAMP receptors are also reported in plants which evolves a common evolutionary origin of pathogen defense systems in higher eukaryotes (Nürnberger and Brunner, 2002). Many times, the pathogens overcome the MTI/PTI immune response by evolving a strategy and promoting infections through the release of effector molecules into the plant cells resulting in effector-triggered susceptibility (ETS). The effector molecule activates ETI, the *R*-gene based vertical immunity, an amplified version of PTI which induces hypersensitive cell death (HR) (reviewed in e.g., Hogenhout et al., 2009; Muthamilarasan and Prasad, 2013). Typically, pathogen spread is prevented by the activation of a single NB-LRR receptor by one pathogen effector (directly or indirectly) to establish immunity. The direct and indirect interactions between *R* and *Avr* gene products is well documented (Keen, 1990; Jia et al., 2000; Dodds et al., 2006). The effector molecules have specific targets in the host and this effector perception mechanism is explained by Guard Model (Van der Biezen and Jones, 1998; Dangl and Jones, 2001). Over the past years, new findings of the indirect recognition of effectors are inconsistent according to Guard Model. It is now well established that multiple targets in hosts are present for different pathogen effectors and the classical Guard Model does not explain this if the plants lack the R protein (van der Hoorn and Kamoun, 2008). Basically, the Guard Model proposed that in the presence of a functional *R* gene, the pathogen perceptions enhanced while in the absence of a functional *R* gene, the pathogen perceptions decrease. This is an evolutionarily unstable situation which can be better explained by Decoy Model (van der Hoorn and Kamoun, 2008), outline the concept of “decoy” which mimics the effector targets to trap the pathogen into a recognition event. The Decoy Model implies that the effector target acts as a decoy which is monitored by the R protein and function on pathogen perceptions even in the absence of its cognate R protein (van der Hoorn and Kamoun, 2008). NB-LRR-mediated immunity is the main line of defense against adapted pathogens that effectively blocks PRR-mediated immunity via effector proteins (review in e.g., Jones and Dangl, 2006; Wirthmueller et al., 2013). Systemic acquired resistance (SAR) is activated at the infection site which halts the progress of infection to the unharmed tissues by the activation and expression of pathogenesis-related (PR) proteins (Durrant and Dong, 2004;Van Loon et al., 2005; Park et al., 2007). The advances in genomics tools such as dual RNA-seq of plants and pathogens and the role of non-coding RNAs (ncRNAs) in regulating the plant defense responses have further enhanced our understanding in simultaneous comparative data analysis of both plants and pathogens and defense responses (Westermann et al., 2012, 2016; Meyer et al., 2016). The NGS technique like dual RNA-seq for the simultaneous study of host and pathogen transcriptomes during their interaction is probe independent and can be easily adopted for any plant-pathogen interaction study is an unbiased approach which along with detection of differentially expressed genes also identify the changes in transcriptional regulatory events (Westermann et al., 2012; Mardis, 2013; Das et al., 2015; Enguita et al., 2016). Similarly, non-coding RNAs like microRNAs, phasiRNAs and long intergenic non-coding RNAs (lincRNAs) are very important players in plant responses to different pathogens and polish up the innate immunity mechanism like PAMP- and effector-triggered defense responses of plants. The study of these non-coding RNAs showed that how the epigenetic effects regulate the plant genes involved in defense against pathogens (Enguita et al., 2016; Lenandias-Briere et al., 2016; Meyer et al., 2016).

Other than microbial (or pathogen) associated molecular patterns (MAMP/PAMP) triggered immunity (MTI/PTI) and ETI, many research findings suggested the phenomena of *trans*-generational immune memory in plants. This means that the immune memory is transferred to the subsequent generation. This trans-generational immune memory in plants has been studied for the environmental stresses and upon challenging the plants with pathogens (Slaughter et al., 2012). Mainly it leads to effective adaptations to that particular stress in plants in next generation referred as acquired immune power (Molinier et al., 2006; Jaskiewicz et al., 2011; Muthamilarasan and Prasad, 2013). When *Arabidopsis* is challenged with an avirulent strain of *Pseudomonas syringae*, the next generation plant showed an immediate and increased accumulation of Salicylic acid (SA) signaling pathway transcripts with enhanced disease resistance (Luna et al., 2012; Slaughter et al., 2012). This type of immunity in plants also suggested the ability of plants to inherit the resistance to the next generation.

The plant-pathogen interactions and plant immunity have always been an important aspect of research and lead to some interesting information like adaptation of unique immune mechanisms of plants against pathogenic strains, R-protein-mediated action, siRNA silencing, post-transcriptional silencing (PTGS) involving cellular RNAs and *trans*-generational immune memory (Voinnet, 2008; Muthamilarasan and Prasad, 2013). In spite of many remarkable discoveries in the field of plant immune system, few mysteries are still undeciphered like identification of many *Avr* genes involved in plant-pathogen interactions, plant root immune mechanisms, molecular mechanisms of colonization pathogens in plants, regulation of cellular activity and gene expression, signaling mechanisms involved in plant immune response. Therefore, further advancement in post-genomic era technologies will pave the way to better understand the plant-pathogen interactions and plant immunity.

## Beneficial Plant Microbe Interactions and Its Relevance

Today, the world faces a continuous challenge to feed the ever growing world population as nearly 1 billion people go hungry every day (Reid, 2011). Less productivity, limited arable land, and water for irrigation as well the loss of yield due to diseases are the primary reasons behind high demand and low supply of food grains. Use of fertilizers, plant breeding, and genetic engineering approaches are exploited to increase the crop yield, but they are expensive, slow and highly specific and cannot be grown in the different environment and are less practical (Reid, 2011). Thus, it is the need of the hour to look for other approaches other than genetic improvement of plants against the pathogens. Few researchers have brought a different perspective to this and emphasize on the exploitation and harnessing of plant-associated microorganisms which are beneficial to plants and have positive effects on plant–microbe interactions (Farrar et al., 2014). These beneficial plant–microbe interactions can help to feed the world.

In beneficial plant–microbe interactions, plants and microbes developed cooperative and beneficial relationship which helps in improving the host plant resistance to a wide variety of stresses, including diseases, drought, salinity, heavy metals, toxins, nutrient stresses, and extreme temperature (Reid, 2011). This beneficial plant–microbe partnership will also help in increasing the crop productivity at low-cost expenses. In the recent years, this relatively new understudied approach has generated a new hope for the world and can be a part of the new Green Revolution (Reid, 2011).

Plant and microbes both play important roles in the contribution of the beneficial plant–microbe interactions. The well-studied examples are symbiosis, where both plant and microbes are benefited, for e.g., nitrogen-fixing bacteria which survive in root nodules of leguminous plants and form a mutually beneficial relationship (Oldroyd et al., 2011). Arbuscular mycorrhizal fungi (AMF), typically found in all kinds of soils, live within the plant roots and helps in the phosphate absorption from the soil (Smith and Smith, 2011). The addition of AMF in the tropical soils helps in reducing the use of phosphate fertilizers along with the improvement in crop yield (review in e.g., Bonfante and Genre, 2010; Rey and Schornack, 2013; Knief, 2014; Ramalingam et al., 2015). Quorum sensing and biofilm formation enables bacterial populations to adhere to plant tissues and leads to beneficial plant–microbe interactions (Mathesius et al., 2003; Ramey et al., 2004). Many genes have been identified in bacteria, *Bacillus amyloliquefaciens*, for biofilm formation, root colonization, and plant growth promotion (Budiharjo et al., 2014). The bacterial quorum sensing signals (QSS) have the profound effect on plants transcriptome and proteome (review in e.g., Mathesius, 2009). Plant production system optimization and bacterial manipulations for the production of higher yield in plants are targeted by biofilm formation and quorum sensing which enables the bacterial population to adhere the environmental surfaces including plant tissue, cell to cell adhesion between bacteria and the response of plants to bacteria QSS (Farrar et al., 2014). Some microbial product, mainly the bacterial enzyme takes part in the protection of host plants against a variety of stresses, like drought and flooding, high salinity, heavy metals and also against pathogens. One interesting way to evade the drought stress in plants is to produce more trehalose which helps in stabilizing the membranes and enzymes. Instead of biotechnological engineering of plants to produce more trehalose, it would be more effective to use bacteria which will provide the surplus trehalose in association with plants. Beneficial endophytes, the microbes which live within the plants without eliciting any disease response, in some way involved in triggering the plant induced systemic resistance (ISR) against some pathogenic bacteria (Kloepper and Ryu, 2006). The advent of NGS technologies and other molecular tools, like complete genome sequencing, metagenomics, transcriptomics, proteomics and fluorescent tagging, and localization studies are of great use in deciphering the biological functions and beneficial plant–microbe interaction studies.

The different classes of plant–microbe interactions elicit different response mechanism. A special class of plant-bacterium interaction is *Agrobacterium tumefaciens*-Plant interaction: a biotrophic interaction which, unlike pathogenic fungi or bacteria does not produce disease symptoms, but rather alters the physiology and morphology of infected host plants (Pitzschke, 2013). Research and significant findings in the past four decades of *Agrobacterium*-Plant interaction proved an excellent paradigm to understand different aspects of plant-bacterium interactions (Gelvin, 2003; Brencic and Winans, 2005; McCullen and Binns, 2006; Yuan and Williams, 2012; Pitzschke, 2013; Subramoni et al., 2014). The recent developments in *Agrobacterium tumefaciens* responses to various plant-derived signaling molecules which help in its pathogenicity and the activation of virulence genes induces the transfer and integration of T-DNA from its Ti-plasmid into the plant nucleus (Subramoni et al., 2014). Many published research articles hinted a hierarchical activation of virulence resulting from the response of combinations of plant-derived chemical signals, like *Agrobacterium* responses to acidic signals (Winans, 1992; Yuan et al., 2008a,b; Wu et al., 2012), plant-derived phenolic compounds (Dixon and Paiva, 1995; Zhu et al., 2000; Brencic et al., 2004; Cho and Winans, 2005) and plant-derived sugars in the rhizosphere (Peng et al., 1998; Nair et al., 2011; He et al., 2009; Hu et al., 2013). Many plant-derived signals and plant hormones, possibly function additively and act in concert and play negative roles in the modulating *Agrobacterium* virulence, Ti plasmid copy number, and quorum sensing (Yuan et al., 2008a; Subramoni et al., 2014). Basically, the infection of *Agrobacterium* into the plants is a means to convert plant cells into a factory to secure nutrients and maintain the genetic integrity of nature (Subramoni et al., 2014).

The importance of *Agrobacterium tumefaciens* infection and transformation success has always been an important aspect of research for both the microbiologists and plant scientists. The success of transformation largely depends on the host defense mechanisms and recalcitrance to *Agrobacterium*-mediated transformation (Nurnberger et al., 2004; Boller and He, 2009; Pitzschke, 2013). Various defense components like MAPKs, defense gene expression, production of reactive oxygen species (ROS) and hormonal adjustments and its manipulation by the *Agrobacterium* decides whether the transformation will occur or not (Li et al., 2005; Yuan et al., 2007; Anand et al., 2008; Lee et al., 2009; Pitzschke et al., 2009; Vellosillo et al., 2010; Zhao et al., 2011). Various biotechnological approaches have been reported to overcome or improve the transformation efficiency in recalcitrant plants, like the use of modified *Agrobacterium* strain, modification in plant growth media/conditions, targeted manipulation of host plants and detailed knowledge of plant-*Agrobacterium* interactions (Pitzschke, 2013). Thus, the *Agrobacterium* pathogenicity and successful transformation efficiency largely depend on particular recognition, response and adjustment to chemical signals which are plant-derived and further to hormones which lowers the defense level in plants.

## Genome-Scale Modeling to Study Plant-Microbe Interactions at Metabolic Level

In recent times, we have a better understanding of plant-pathogen systems at the molecular level, along with the multifaceted signaling pathways which otherwise coordinate different defense responses in plants. Despite better knowledge and a huge amount of available data and information gathered during the genomic and post-genomic era, there is hardly enough research focused at molecular level due to the high cellular complexity and interactions of cellular components to large numbers of internal and external conditions (Collakova et al., 2012). Clearly, molecular studies have advanced but the regulation and changes to the plant metabolism during pathogen attacks have been recently emerging. Various new and sophisticated methods are coming up to study plant metabolites. Genome-scale modeling (“GEMing”) is used to mathematically to model the metabolism and it is basically an *in silico* metabolic flux model which has been derived from the currently available genomic data. Genome-scale models are becoming quite a challenge to exploit to analyze the phenotype during host–pathogen interactions (Collakova et al., 2012).

The information gathered from the available genomics, transcriptomics, proteomics, and metabolomics tools are basically required for the metabolic network modeling of plants and pathosystems. Till now many plant-bacterial and plant-fungus pathosystems have been extensively studied which are listed in the table below (**Table 1**). The genome-wide study of the interaction between plants and its related pathogens has been possible in recent years because of the advancement in genome sequencing and annotations (Kanehisa et al., 2002; Krieger et al., 2004; Shendure et al., 2004). The complete genome sequence helps in the genome-wide annotations of all the proteins, enzymes and other related metabolic reactions (Duan et al., 2013). The merging of the metabolic network of plants and their related pathogens proved to be important in the study of negative and positive effects of joint metabolic networks (Duan et al., 2013). The study of metabolic networks of mentioned plant-pathogen pairs showed that the impairment pattern is largely determined by the pathogens. No strong segregation was also evident at the kingdom level (for bacteria and fungi) (Duan et al., 2013).

**Table 1:** Overview of selected plant–microbe pairs extensively studied and represented as most suitable pathosystem.

S. No	Pathogen	Plant	Pathogen type	Reference
1	*Pseudomoas syringae* pv. *Tomato*	*Arabidopsis thaliana*	Bacterium/hemi-biotrophic	De Vos et al., 2005; Glazebrook, 2005
2	*Hyaloperonspora parasitica*	*Arabidopsis thaliana*	Oomycetes	Slusarenko and Schlaich, 2003; Rentel et al., 2008
3	*Phytophthora infestans*	*Arabidopsis thaliana*	Oomycetes	Huitema et al., 2004
4	*Xanthomonas oryzae* pv. *Oryzae*	*Oryza sativa*	Bacterium/biotrophic	Hammond-Kosack and Jones, 1997; Salzberg et al., 2008
5	*Ustilago maydis*	*Zea mays*	Fungus/biotrophic	Mueller et al., 2008
6	*Melampsora larici-populina*	*Populus trichocarpa*	Fungus/biotrophic	Hacquard et al., 2012
7	*Sclerotinia sclerotiorum*	*Glycine max*	Fungus/biotrophic	Malencic et al., 2010; Amselem et al., 2011
8	*Magnaporthe oryzae*	*Orzo sativa*	Fungus/biotrophic	Bryan et al., 2000; Orbach et al., 2000; Skamnioti and Gurr, 2009; Yoshida et al., 2009

Earlier plant pathosystems were best studied by one-gene-at-a-time or a one-protein-at-a-time approach, but as more and more genome sequence of hosts and pathogens are coming up, a more holistic approach was started to study plant-pathogen interactions. At the beginning of this decade, the transcriptomic tools like CDNA microarrays, SuperSAGE gene expression profiling have been developed to study signaling in *Arabidopsis thaliana-Pseudomonas syringae* pv. *Tomato* and *rice-Magnaporthe oryzae* interactions (Matsumura et al., 2003; de Torres-Zabala et al., 2007). Later on, new generation sequencing technologies have also been used for transcriptional profiling by RNAseq to study plant-pathogen interactions. Along with transcriptomic, proteomic technologies like 2D gels, MS/MS, iTRAQ proved as important tools to dissect plant-pathogen interactions (Jones et al., 2004). Then came the post-genomic era tools which are now used to validate the biologically and functionally obtained results which were fetched during the genomics era. The very objective of the post-genomic era is to link the sequences to phenotypes and to better understand the interactions between plant and pathogens (Huitema et al., 2004).

The various ‘omics’ network requires the use of kinetic information. Now various approaches which do not require kinetic information like metabolic network reconstructions, Genome-scale reconstructions, and targeted metabolic reconstructions were employed to study the biological processes at metabolic and regulatory levels (Pinzon et al., 2010). The first bacterial genome sequence completion of *Haemophilus influenza* in 1995 has opened a new avenue of research for the construction of a computational model of an organism that envisages its complete operation from genome sequence alone (Seaver et al., 2012). Later on, development of ‘virtual plant’ in 2000 for *Arabidopsis* has paved the way for its use at a higher level. A detailed knowledge of the function of all the genes, their interactions, and the cooperation between different genes to drive and sustain the life of a multicellular organism are required to develop a genome-scale model of a whole organism (Seaver et al., 2012). In the post-genomic era, the important achievement is the progress in sequencing, annotation, reconstruction and modeling *in silico* the metabolic networks of the entire organism (Seaver et al., 2012). Genome-scale metabolic modeling is a concept which investigates the metabolic capabilities and drawback of an organism (Plants/pathogens) largely on the basis of proteins/enzymes and transporters encoded by its genes. A metabolic model has been useful in, like, elucidation of physiology and metabolism, reaction compilation, importance of metabolic reaction steps, gene-protein-reaction (GPR) associations, localization, directionality, and reversibility of reactions and to design rational metabolic engineering strategies (Kauffman et al., 2003; Murabito et al., 2009; Schilling et al., 2000; Liu et al., 2010; Senger, 2010; Collakova et al., 2012; Seaver et al., 2012). Genome-scale model have been successfully implemented and developed for many organisms, including bacteria, fungi, plants, and animals because of the advancement and up gradation of high-throughput sequencing, proteomics, and metabolomic technologies (Famili et al., 2003; Duarte et al., 2007; Feist et al., 2009; Fu, 2009; Baumler et al., 2011; Orth et al., 2011; Rolfsson et al., 2011). Till now, for plants the genome-scale metabolic models are only available for *Arabidopsis*, barley (*Hordeum vulgare*), maize (*Zea mays*), sorghum (*Sorghum bicolor*), sugarcane (*Saccharum officinarum*), rape seeds, and *Arabidopsis* genoome-scale model (AraGEM), and C(4) genome-scale model (C4GEM) (Poolman et al., 2009; Grafahrend-Belau et al., 2009; Dal’Molin et al., 2010; Rolletschek et al., 2011; Hay and Schwender, 2011a,b; Pilalis et al., 2011; Saha et al., 2011). The main aim of the metabolic network reconstruction model is to consider all metabolic interactions activated during plant-pathogen interactions. The genome-scale reconstruction model (GSRM) is based on the metabolic reconstruction at the genome scale for the analysis and interpretation of metabolite concentrations and the reactions of the metabolic state of the cell at a given time under specific conditions (Carrera et al., 2009; Durot et al., 2009). Targeted metabolic reconstructions are basically an extension of transcriptomic research which provides an important information of a particular phenotype network under specific conditions (Pinzon et al., 2010). GSRM has been developed for many organisms and its application is widely important in interpretation of high-throughput data, system metabolic engineering in which whole-cell networks and systems-level analyses are used to optimally engineer the whole cell, discovery and identification of new hypothesis on the basis of existing hypothesis, to understand the multi-cellular communities interactions for the phenotype-genotype gap bridging, and to investigate functional evolution of metabolic and regulatory networks (Oberhardt et al., 2009). GSRM enabled analysis of emergent phenomenon by focussing on entire networks rather than individual pathways or genes, and many computational techniques have been described to explore network properties. These types of network-level analysis will be significant to completely disentangle the complex genotype-phenotype relationships in cells (Oberhardt et al., 2009).

## Pathogen-Host Interaction Database (PHI-base)

The pathogens are the most notorious creatures on this earth, evolving rapidly and constantly, causing diseases and threatening the plants as well as animal health. The pathogen-host interaction database (PHI-base), established in 2005 is molecular and biological information, catalogs of genes which directly affects the consequences of host–pathogen interactions (Winnenburg et al., 2008). PHI-base is a multi-species, web-based database which helps in the experimental verification of pathogenicity data, virulence and effector genes from bacterial, fungal and oomycete pathogens (Winnenburg et al., 2008; Urban et al., 2015). PHI-base also includes plant endophytes. PHI-base has proved to be an important tool in which genes of agronomically important pathogens are discovered and can be potential targets for chemical intervention and host modification. There are two categories in which PHI- base can be used, i.e., academic and non-academic. Sixty percent of the species within PHI-base are represented by plant pathogens. Easy to use and hi-tech search tools which allow users to know more about PHI-base can directly be fetched at www.phi-base.org. Larger comparative biology studies, approaches to systems biology, higher annotation of genomes, proteome and transcriptomes data sets, all are enabled in flat file downloads. Since 2014, www.phytopathdb.org has displayed all the related information regarding phenotype from PHI-base (Kersey et al., 2014). The international community is often approached by PHI-base for delivering best searching and sorting tools so the pathogen-host interaction studied could be made easy.

The latest version, PHI-base 3.8, stores information on 3562 pathogen genes, 4954 plants, and animal interactions identified from 1243 references (Urban et al., 2015). The phenotypes and gene function information were obtained manually by retrieving from peer-reviewed literature.

## Emerging Phytopathogens: The Current Challenges In Plant-Microbe Interactions

Plants like humans are frequently attacked and challenged by an array of benefits and pathogenic microorganisms. Many pathogens invasions which cause disease are halted by the defense mechanisms of plants, but the emergence and evolution of newly faced pathogens may help them in escaping the solid host innate immunity (Stukenbrock and Bataillon, 2012; Misra and Chaturvedi, 2015). Both plants and pathogens evolve in response to each other and this co-evolutionary arms race and agricultural practices lead to pathogens invasion and colonization in the new host in native communities in which they have no prior evolutionary history (Britton and Liebhold, 2013; Misra and Chaturvedi, 2015). The catastrophic outbreaks of the exotic pathogens in an ecosystem are driven by the increase in human population, human interference, the increase in global trade frequency and co-evolution of both host and pathogens. The emerging pathogens lead to the emergence of plant disease and the main reason behind this are, the introduction of evolved pathogens or new pathogen species, human migration, divergence, speciation, plant susceptibility and abundance, hybridization among existing pathogens and of course the climate change (Garrett et al., 2010; Misra and Chaturvedi, 2015). For the past few decades, the emergence of new pathogens is more frequent due to above-mentioned reasons which lead to the extinction of many wild species, biodiversity loss and yield in crop production (Cobb et al., 2012; Fisher et al., 2012). Therefore, it is the need of the hour to understand and identify the emerging pathogens and develop strategies to counter them. The recent reviews on the emergence of pathogens (**Table 2**) have created a lot of interest in the field of plant-pathogen interactions among the plant pathologists (Anderson et al., 2004; Misra and Chaturvedi, 2015; Thynne et al., 2015).

**Table 2:** List of emerging phytopathogens and strategies to manage the affects of emerging pathogen.

Emerging Pathogens	Host Plant	Strategies to manage the affect of emerging pathogens	Reference
**Bacteria**			
*Burkholderia glumae* *Dickeya solani*	Rice Potato	(1) A deeper insights and understanding in cross-kingdom horizontal gene transfer (HGT). (2) Knowledge of shared genes and proteins among the genomes of pathogens. (3) A newer insights into phage-mediated regulation of bacterial pathogenesis.	Toth et al., 2011; Sharma et al., 2013; Varani et al., 2013; Thynne et al., 2015
			
**Fungus**			
*Ramularia collo-cygni*	Barley	(1) Development of new approaches to explore genomic data and to characterize patterns of natural selection like, genome evolution, recombination pattern, and population dynamics. (2) More emphasis should be in the study of nucleotide diversity and analysis of repeat-rich regions. (3) Population genomic analysis, particularly identification of strongly selected genes, evolutionary potentials. (4) A deeper insights and understanding in cross-kingdom HGT.	Leonard and Bushnell, 2003; Solomon et al., 2006; Walters et al., 2008; Bahri et al., 2009; Ma et al., 2010; Husson et al., 2011; de Jonge et al., 2012; Kjær et al., 2012; Raffaele and Kamoun, 2012; Stukenbrock and Bataillon, 2012; Stukenbrock et al., 2012; Maciel et al., 2014; Misra and Chaturvedi, 2015
*Melampsora columbiana*	Poplar rust		
*Verticillium longisporum*	Wilt in many plants		
*Verticillium dahlias*	Wilt in many plants		
*Botrytis allii*	Onion		
*Puccinia striifornis*	Wheat		
*Stagonospora nodorum*	Wheat		
*Hymenoscyphus pseudoalbidus*	Ash tree		
*Fusarium graminearum*	Cereal crops		
*Chalara fraxinea*	Ash tree		
*Zymoseptoria pseudotritici*	Grass species		
*Magnaporthe oryzae*	Wheat		
*Neurospora crassa*	Bread		
*Mycosphaerella graminicola*	Wheat		
			
**Oomycetes**			
*Phytophthora ramorum*	Oak	(1) A deeper insights and understanding in cross-kingdom HGT. (2) Analysis of evolutionary dynamics. (3) Continuing genome sequencing and pathogenomics research. (4) Effectoromics and plant host responses. (5) Genome-wide cataloguing of oomycete effectors.	Rizzo et al., 2005; Baxter et al., 2010; Raffaele et al., 2010; Goss et al., 2011; Links et al., 2011; Richards et al., 2011; Grünwald et al., 2012; Misra and Chaturvedi, 2015
*Phytophthora alni*	Alders		
*Phytophthora andina*	Potato		
			
**Virus**		(1) A system biology approach to study their interaction with plants with the help of collection and analysis of high-throughput molecular data and omics-scale data.	Hanssen and Thomma, 2010; Kikuchi et al., 2011; Hwang et al., 2012; Misra and Chaturvedi, 2015

The emergence of pathogens has always been a serious threat to agricultural practices, food security, and conservation of plant species. It is now very important to identify new pathogens and understand how they emerge (Thynne et al., 2015) and the timely management strategies to halt the invasion of emerging pathogens. The plant-pathogen interaction is a complex process and the emergence of new pathogens has always been a challenge for the scientist’s world over. Trade and man-made movements in plant and plant based products have significantly affected the global distribution and diversity of plant pathogens. Elucidation of migration pathways can be used to scrutinize the movement of pathogens for efficient disease management or quarantine measures (Goss, 2015). Genomics-based genetic marker discovery is permitting unique collection of population genetic data for plant pathogens. The ever increasing genome sequences of phytopathogens have improved our understanding of accelerated genome adaptation and ability to cause plant disease by pathogens (Benson et al., 2006, 2012; Thynne et al., 2015). Accelerated genome adaptation is the process in which the pathogens adapt themselves to the new environment and changes pathogenicity pattern. The most widely studied and popular mechanism of accelerated genome adaptations is horizontal gene transfer (HGT) and inter-specific hybridization (Stukenbrock et al., 2007; Keeling and Palmer, 2008; Giraud et al., 2008; Raffaele and Kamoun, 2012). The two mechanisms have been comprehensively reviewed (review in e.g., Misra and Chaturvedi, 2015; Thynne et al., 2015). The ongoing research on the emergence of new pathogens and its impact on pathogenicity have created new ideas to counter these emerging pathogens. The population genomics study can be an important tool to understand the adaptive evolution of plant pathogens for the development of improved disease management strategies. Other strategies like popularization of agricultural heterogeneity and restriction in the transfer and movement of plant materials will also help in the management of emerging plant pathogens. Moreover, better communication among the plant pathologists, ecologists, epidemiologists, many academic researchers and other related partners will facilitate the ongoing research for successful management of emerging phytopathogens.

## Conclusion and Perspectives

**FIGURE 2:**
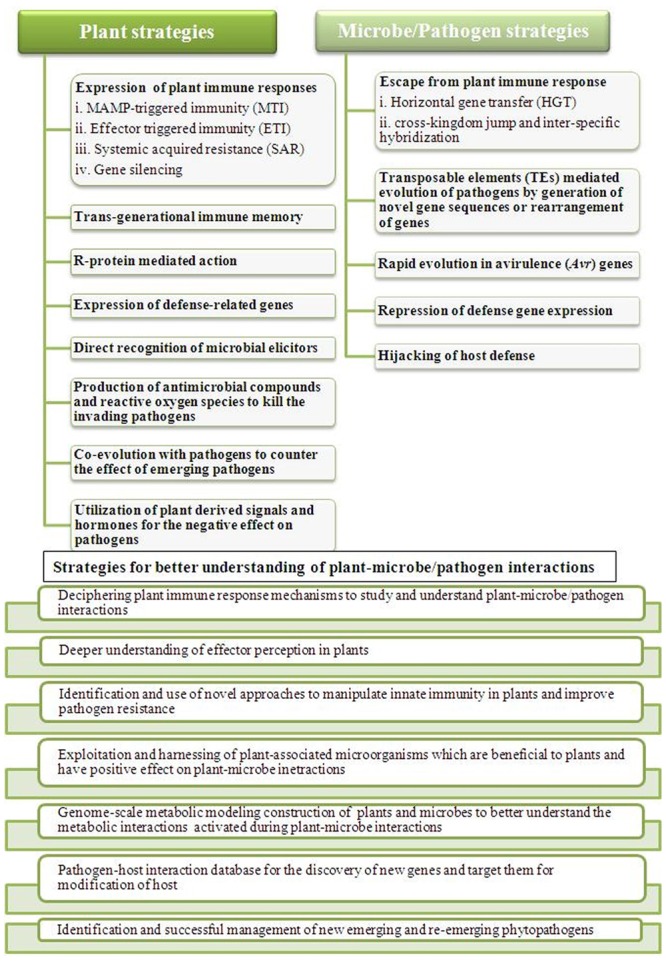
**Outline of plant–microbes/pathogens responses during their interactions and listing some of the important strategies to counter them.** Plants as well as microbes/pathogens both faces challenges of each other during the battle and to better understand their interactions for effective outcome, different strategies needs to be implemented to address the problem of global food security.

Our understanding of responses during the plant–microbe interactions has taken a big leap forward. However, we still have several aspects and problems to overcome during this decade to get closer to answering the various questions related to these interactions for the development of pathogen-resistant crops for human sustainability (Misra and Chaturvedi, 2015; Thynne et al., 2015). Our knowledge is partial towards the stress responses in plants as a whole against pathogens. Various biotechnological approaches and their continuous advancement will be required to collect and integrate the information into a complete picture (Knief, 2014). In this area of research, the near future faces many challenges which have to be met for the integrated knowledge of plant-pathogen interactions. The battle between plants and pathogens have always been exciting, informative and challenging (**Figure 2**). These challenges are mainly, identification of sensors and signaling pathways involved in the interactions, understanding the molecular basis of interplay among various types of stresses and response, identification of key factors involved in such interactions mainly during plant immune responses, understanding the progression of signals and disease to other parts of plants, long-term response of plant under the pathogen attack in nature, identification and successful management of new emerging and re-emerging phytopathogens and development of pathogen-resistant crops. Over the last few years, biomolecular research has progressed from the completion of the genome sequencing project of many plants and pathogens to functional genomics and the application of this knowledge to advance our understanding of their interactions and disease management (Knief, 2014). It is clear that genomic information alone, although crucial, is not sufficient to completely explain these intricate interactions between plants and pathogens (Bender, 2005). The next-generation sequencing technology along with various ‘omics’ technologies and development of databases and metabolic modeling are closing the gaps and bringing together the microbial ecology and molecular plant pathology to better understand the plant immunity and pathogen virulence and development of ideas. It is a tough task but not at least impossible to have a better understanding of plant–microbe interactions, so that better strategies can be implemented and the problem of global food security can be solved.

## Author Contributions

All authors listed, have made substantial, direct and intellectual contribution to the work, and approved it for publication.

## Conflict of Interest Statement

The authors declare that the research was conducted in the absence of any commercial or financial relationships that could be construed as a potential conflict of interest.
